# A new glance at autophagolysosomal-dependent or -independent function of transcriptional factor EB in human cancer

**DOI:** 10.1038/s41401-023-01078-7

**Published:** 2023-04-03

**Authors:** Ting Wang, Yi Qin, Zeng Ye, De-sheng Jing, Gui-xiong Fan, Meng-qi Liu, Qi-feng Zhuo, Shun-rong Ji, Xue-min Chen, Xian-jun Yu, Xiao-wu Xu, Zheng Li

**Affiliations:** 1 Department of Pancreatic Surgery, Fudan University Shanghai Cancer Center, Shanghai, 200032 China; 2 Department of Oncology, Shanghai Medical College, Fudan University, Shanghai, 200032 China; 3 Shanghai Pancreatic Cancer Institute, Shanghai, 200032 China; 4 Pancreatic Cancer Institute, Fudan University, Shanghai, 200032 China; 5 Department of Hepatobiliary Surgery, The Third Affiliated Hospital of Soochow University, Changzhou, 213000 China

**Keywords:** neoplasm, autophagy-lysosomal system, TFEB Protein, Mammalian Target Of Rapamycin Complex 1, tumor progression, metastasis

## Abstract

Autophagy-lysosome system plays a variety of roles in human cancers. In addition to being implicated in metabolism, it is also involved in tumor immunity, remodeling the tumor microenvironment, vascular proliferation, and promoting tumor progression and metastasis. Transcriptional factor EB (TFEB) is a major regulator of the autophagy-lysosomal system. With the in-depth studies on TFEB, researchers have found that it promotes various cancer phenotypes by regulating the autophagolysosomal system, and even in an autophagy-independent way. In this review, we summarize the recent findings about TFEB in various types of cancer (melanoma, pancreatic ductal adenocarcinoma, renal cell carcinoma, colorectal cancer, breast cancer, prostate cancer, ovarian cancer and lung cancer), and shed some light on the mechanisms by which it may serve as a potential target for cancer treatment.

## Introduction

Macroautophagy (hereafter referred to as autophagy) is an intracellular lysosomal degradation pathway that is a core catabolic pathway in all eukaryotes and is indispensable to normal cell activity [1]. In addition to the induction of autophagic flux to sustain metabolic homeostasis and survival, cells depend on autophagy for maintaining homeostasis under physiological conditions [2–4]. Abnormal stimulation, including extracellular stress and intracellular stress (e.g., metabolic stress, hypoxic stress, oxidative stress, mechanical stress, immune signals, overcrowding, and protein aggregation) [5–10], activates or attenuates autophagy, which leads to the initiation and progression of multiple pathological processes and diseases [11].

Interestingly, autophagy plays dual context-dependent roles in cancer: autophagy has been found to prevent cancer and promote the growth and metastasis of tumors [12–15]. For example, research has indicated that autophagy can prevent tumor growth in the early stages, namely, during tumorigenesis, through the degradation of toxic debris [12, 16–18]. However, after tumors form, autophagy can promote their growth and survival by resolving challenges caused by diverse stresses [19–21]. In addition, autophagy in noncancer cells, which is called host autophagy, can lead to cancer progression [22].

Autophagy proceeds through four sequential steps, including the formation of a double-layered membranous precursor of autophagy; the extension of this membrane structure, the autophagosome, which encapsulates targeted cell components; closing of autophagosome and its binding to a lysosome, which degrades the autophagosome contents, many of which are recycled [11, 23–25].

Because the autophagosome fuses with lysosomes to expose the contents to the enzymes and reactive oxygen species (ROS) that degrade them, lysosomes are indispensable to the complete autophagic process [26]. Therefore, the destruction of lysosomal function and structure, a decrease in lysosome number, attenuation of lysosomal protease activity, or inhibition of autophagosome–lysosome fusion leads to impaired autophagic flux [27, 28].

Lysosomes are membrane-bound degradative compartments discovered by Christian de Duve [1, 29], and more than 50 lysosomal enzymes have been identified to date [30]. V-ATPase maintains the acidic internal pH between 4.5 and 5.5 within the lysosome, which enables the function of luminal hydrolases for the degradation of cellular components [31, 32]. In addition to their role in degradation, other functions have recently been attributed to lysosomes; for example, they are signaling hubs that establish contacts between cellular organelles and transmit signals, which means that lysosomal dysfunction is critical for the onset and progression of many more human diseases than we previously thought [33–38].

Autophagy is a tightly regulated process mediated by the PI3K-AKT-mTORC1 signaling pathway [2]. In addition, other regulators, such as members of the MITF family, AMPK, Wnt, p53, PtdIns3P, microRNAs/miRNAs, etc., function in autophagy [39–41]. Lysosomes are regulated by a sophisticated system to meet cellular needs when the microenvironment changes; that is, they have multiple functions, and lysosomal proteins are not merely constitutively transcribed from housekeeping genes [42]. Because lysosomes and autophagy machinery are both sophisticated and intimately related, the number of studies directed to the core regulators of the autophagolysosomal system is constantly increasing.

TFEB is a member of the MiT/TFE family of transcription factors and is the master regulator of lysosomal biogenesis and autophagy, exerting its effect by directly binding to the promotor of lysosomal and autophagic genes [43]. Since the function of the autophagolysosomal system has been shown to be globally coordinated through transcriptional regulation, lysosomal genesis and its functional activation and the many steps in autophagy share the same sequence in the promoters of key corresponding genes: the coordinated lysosomal expression and regulation (CLEAR) motif. TFEB binds to a CLEAR motif and promotes the expression of the corresponding gene. Thus, it directly promotes the activation of lysosomal protein production and is thereby critical to multiple steps in autophagy; as a result, TFEB is deemed to be very important [44, 45]. In view of the important role of autophagy in tumorigenesis and progression and the higher rates of autophagy found in an increasing number of cancer types, the role of its important regulatory factor TFEB in cancer has also received increasing attention. TFEB promotes cancer onset and progression by regulating the activity of the autophagolysosomal system. In addition, researchers have also found that TFEB regulates the acquisition of pro-tumor phenotypes in an autophagolysosome-independent manner, and this important function has been widely associated with the development and progression of various types of tumors [46, 47].

In this review, we discuss how TFEB functions in several types of cancer by regulating lysosomal biogenesis and autophagy or other noncanonical mechanisms, even those realized in an autophagolysosome-independent manner. Moreover, we suggest a strategy to fight human cancer by leveraging the TFEB function.

## History of TFEB in the autophagolysosomal system

### Lysosomal activities and autophagy in human cancer

#### The role of the autophagolysosomal system in normal cells and cancer

Through the autophagolysosomal system, damaged and toxic components are degraded, revitalizing cells by recycling nutrient sources that facilitate their responses to several stress conditions and thus promoting their homeostasis and survival [48]. In addition to the autophagolysosomal system promoting health and metabolic balance, it also provides cancer cells with energy and nutrients and is associated with several hallmarks of cancer, creating challenges to cancer treatment [49, 50].

Undoubtedly, lysosomal dysfunction leads to the accumulation of toxic substrates and diseases such as cancer, neurodegenerative diseases, and inherited diseases [51, 52]. However, lysosomal dysfunction in cancer cells interrupts the nutrient supply, starving them of the high level of energy sources they need [53].

#### Autophagy‒lysosome system as a target for treatment and diagnostics

Considering the important role that the autophagy‒lysosome system plays in oncogenesis, targeting the autophagy‒lysosome system has long been recognized as a promising potential treatment strategy and has been reported in several clinical trials in recent years [18, 25, 40, 54–56]. However, research on drugs targeting the autophagy machinery is in its infancy, and one of the only autophagy-targeted compounds, chloroquine (CQ), that has entered clinical research inhibits autophagy by causing lysosomal deacidification and hindering the binding of autophagosomes to lysosomes [57]. Due to its limited selectivity, CQ targets many characteristics of lysosomes, including essential functions, at the same time, which highlights the importance of researching specific targets in the autophagy-lysosomal system [57]. Recently, our group showed that Hernandezine (Her) activates the ROS/AMPK signaling pathway in a concentration-dependent manner and thus induces autophagic cell death in human pancreatic cancer cells [58]. This study provided support for the application of autophagic drugs to treat pancreatic cancer, demonstrated the importance and feasibility of autophagy-related cancer therapies, and opened the door to research into therapeutic strategies by regulating key autophagy pathways.

### TFEB mediates the link between autophagy and lysosomal biogenesis

#### TFEB in the MiT/TFE family of transcription factors and the regulation of TFEB activity

**Fig. 1:**
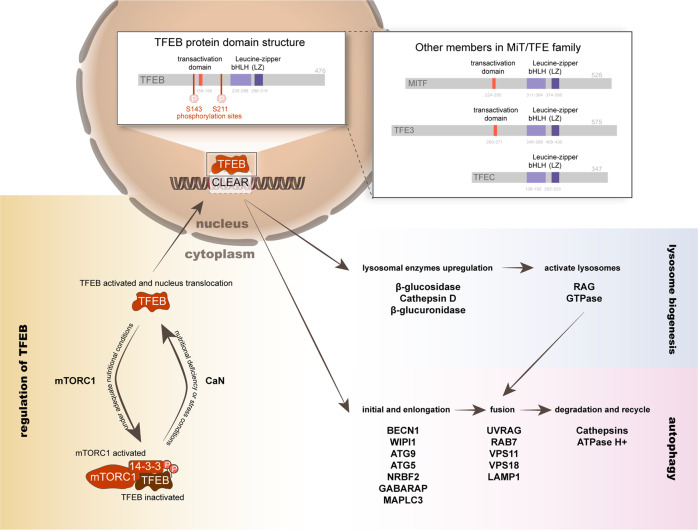
The domain structure of TFEB and its family members, and the working model of TFEB. TFEB is a member of the MiT/TFE family, sharing the same bHLH-Zip domain with other members (MITF, TFE3, and TFEC) of the family. Another domain binds DNA and enables homo/heterodimer formation, thereby activating TFEB and its downstream factors. The activity of TFEB is regulated by mTORC1, which controls intracellular localization and protein activity by phosphorylating specific amino acid sites on TFEB, especially S211 and S143; upon phosphorylation, TFEB binds to 14-3-3 and retaining in the cytoplasm. By dephosphorylating TFEB, CaN promotes its nuclear translocation and function. Lysosomal genes are coordinately expressed and centrally regulated. Specifically, TFEB binds to the CLEAR (coordinated lysosomal expression and regulation) region in the promoter of various genes that regulate autophagy and lysosomal genesis to promote the expression of these genes, increase the production of various enzymes in lysosomes and promote lysosomal activation. Moreover, it promotes the autophagolysosomal process by directly affecting the genes required for multiple steps in autophagy.

TFEB is a member of the MiT/TFE family of transcription factors that encodes four genes, MITF, TFEB, TFE3, and TFEC [44, 59]. All four of these MiT/TFE family-encoded proteins share a common structure: a basic DNA-binding domain and a bHLH-Zip (basic Helix-Loop-Helix Zipper) domain, which is involved the formation of homo or heterodimers that are essential to their activation. These specific structures enable MiT/TFE family members to recognize and bind to the CLEAR motif [60–66] (Fig. 1).

TFEB is regulated mainly at the posttranslational level, mostly via phosphorylation [67]. Two main phosphorylation sites, Ser142 and Ser211, determine the activity of TFEB [44]. Under nutrient-sufficient conditions, Ser142 and Ser211 are both phosphorylated, and TFEB remains in the cytosol and is inactive. Lack of nutrients or lysosome dysfunction leads to the TFEB translocation from the cytosol to the nucleus, where it is activated [44, 64, 68].

The mammalian target of rapamycin complex 1 (mTORC1) plays a central role in sensing the cellular state and regulating TFEB activation [42, 63, 69]. The mTORC1/TFEB axis responds to various stimuli in the microenvironment, regulates several aspects of the cellular physiology, and coordinates lysosomal biogenesis and autophagy [63, 70]. mTORC1 integrates biological signals, including nutrient, hormone, growth factor and oxidative stress response signals, and enhances the synthesis of protein nucleotides and lipids, promoting cell proliferation [44, 70, 71]. The transcription factor EB directly binds to the CLEAR motif, which is located at promotors of lysosomal and several autophagic genes to initiate the transcriptional process of lysosomes and promote autophagolysosomal activity. In turn, overexpression of TFEB induces RagD expression, which is a direct transcriptional target of TFEB, recruits mTORC1 to the surface of lysosomes and thus activates lysosomes. The mutual regulatory effect between mTORC1 and TFEB forms a feedback loop that coordinates anabolic and catabolic pathways and links autophagy to lysosomal biogenesis [42–44, 64, 70, 72].

In addition to the mTOR-dependent phosphorylation pathway, several other kinases have been shown to phosphorylate TFEB [73]. Phosphorylation of S142 in TFEB by ERK2 leads to cytoplasmic retention, and ERK2 knockdown promotes TFEB nuclear translocation [43]. GSK3 and AKT also phosphorylate other serine residues in TFEB, which leads to its cytoplasmic retention, and the inhibition of GSK3 or AKT leads to TFEB nuclear translocation [73]. Studies have shown that the tumor suppressor gene p53 also affects the autophagy-lysosomal pathway by regulating the nuclear translocation of TFEB, but the mechanism remains to be studied [74].

Through its regulatory effect at the posttranscriptional level, the tumor suppressor programmed cell death 4 (PDCD4) reduces the level of nuclear TFEB, thereby inhibiting lysosome biogenesis and function [75].

In addition, STUB1 regulates TFEB activity by degrading it. STUB1 preferentially interacts with phosphorylated TFEB, targeting it for proteasomal degradation through the ubiquitin‒proteasome pathway; thus, overexpression of STUB1 results in decreased levels of phosphorylated TFEB and increased TFEB activity [76].

#### Functions of TFEB in autophagolysosomal degradation and other biological processes

TFEB has long been found to be a master regulator of lysosomal biogenesis by directly binding to the CLEAR motif, which is enriched in the promotor of lysosomal and autophagic genes, and drives the expression of the whole CLEAR-carrying gene network, leading to an increase in the number of lysosomes and autophagosomes and promoting autophagosome–lysosome fusion [44, 45, 77, 78]. Overexpression of TFEB leads to an increase in the number of lysosomes and higher levels of lysosomal enzymes and thus higher autophagic flux, which leads to increased catabolic activity [79]. In contrast, impaired TFEB-mediated lysosomal biogenesis results in several diseases [28].

In addition, more biological processes have been shown to be related to TFEB in recent years. For example, TFEB has been shown to play a role in vascular biology by regulating angiogenesis by modulating endothelial cell activity [77, 80].

Evidence shows that TFEB inhibits ferroptosis by upregulating lysosomal protein production, including superoxide dismutase (SOD), which reduces ROS levels [81]. TFEB also inhibits apoptosis in response to DNA repair. Interestingly, the TFEB-mediated antiapoptotic function continues even when lysosomal functions are inhibited, which means TFEB prevents cells from undergoing apoptosis in a lysosome-independent way [82, 83].

**Fig. 2:**
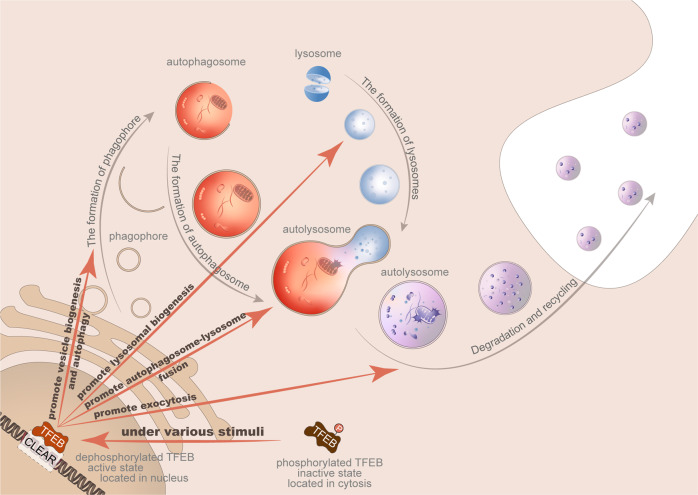
TFEB regulates several processes in lysosomal biogenesis and autophagy. Macroautophagy, also known as autophagy, involves the formation of the double-layered membranous precursor of autophagy, extension of this membrane to encapsulate target ingredients, closing of the autophagosome and its binding to a lysosome, which mediates autophagosome content degradation and recycling. The activation and transcription of TFEB have been shown to play regulatory roles in multiple parts of the autophagy-lysosomal system, including the promotion of vesicle biogenesis, lysosomal biogenesis, autophagosome–lysosome fusion, and exocytosis.

Given the biological processes in which TFEB participates, more researchers are paying attention to the role of TFEB in various diseases, especially cancer, and regarding it as a promising therapeutic target [79, 84] (Fig. 2).

## TFEB as an oncogene and plays a role in the acquisition of several tumoral phenotypes

The rapid proliferation of cancer cells leads to higher demands for energy and synthetic ingredients than needed for normal cells [85]. Therefore, the increase in the activity of the autophagolysosomal system is typical in tumor cells, which makes TFEB a good target for researchers in the cancer field. Compared to that in normal tissues, the upregulated expression of TFEB has been found in several cancers, such as melanoma, renal cell carcinoma, pancreatic adenocarcinoma, non-small cell lung cancer, and colorectal cancer. In contrast, TFEB knockdown leads to the inhibition of tumor growth and fewer cells with malignant phenotypes. The function of TFEB as an oncogene has thus been established in a wide range of cancers [46, 86–93].

There are multiple roles that TFEB plays in cancer onset and progression [67]. Studies indicate that TFEB is related to several tumor phenotypes, including cell proliferation, cell growth, differentiation, migration, and metastasis [30, 46, 91, 94–97].

**Fig. 3:**
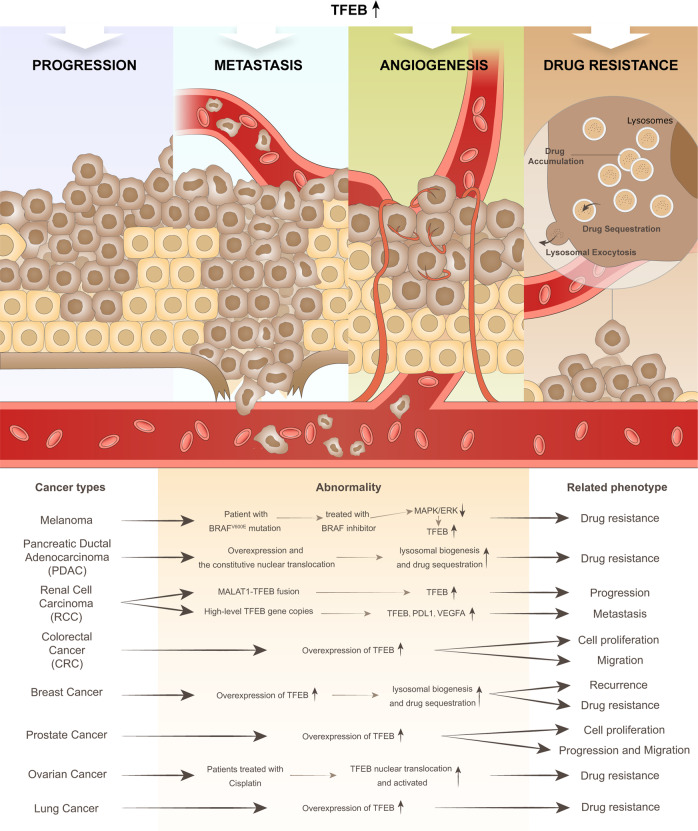
TFEB-related phenotypes and human cancer. Abnormalities in TFEB can induce the acquisition of several cancer phenotypes, such as tumor progression, metastasis, angiogenesis, and drug resistance, and can promote tumor growth through different pathways and mechanisms in different cancer types.

A study showed that increased expression of TFEB is associated with poor prognosis in cancer [95], which demonstrates the value of studying the roles of TFEB in disease and in treatment strategies for various types of cancer (Fig. 3).

### Progression

Overexpression of TFEB is related to the upregulation of metalloproteinases, which are closely related to tumorigenesis [98]. Studies have shown that TFEB promotes cancer progression in several types of cancer via different mechanisms, including lysosomal biogenesis, the epithelial to mesenchymal transition (EMT), cell cycle regulation, and increased invasiveness. [67, 99, 100]. However, it has also been reported that loss of TFEB signaling promotes tumor progression, dissemination, and chemotherapy resistance in melanoma, which indicates that TFEB plays different roles in different types of cancer [101].

### Metastasis

Overexpression of TFEB induces the epithelial-mesenchymal transition and thus promotes the metastasis and invasion of cancer cells in gastric cancer [90]. Silencing of TFEB with specific siRNAs resulted in significant attenuation of cellular metastatic capacity. Interestingly, no particular effect of TFEB silencing has been shown on cell proliferation [95].

### Angiogenesis

In the tumor microenvironment, angiogenesis of quiescent vasculature is continuously activated, which is thought to remain quiescent in normal adult tissues. An abnormally growing vasculature, a hallmark of cancer, provides the necessary nutrients and oxygen for proliferating cancer cells, especially solid tumors, removes metabolic wastes, promoting tumor progression and metastasis [102–104]. Interestingly, TFEB has been reported to be associated with angiogenesis [77].

Studies have shown a direct correlation between TFEB and hypoxia-inducible factor 2-alpha (HIF-2α). Moreover, in addition to HIF-2α overexpression, angiogenesis leading to vessel formation at the leading edge of a tumor is also increased in TFEB-overexpressing cancer tissues [105].

In addition, studies have shown that overexpressed TFEB promotes angiogenesis by increasing the expression of the autophagy gene MCOLN1 (mucolipin-1) at the transcriptional level, as well as activating AMPKα, which is an essential factor for activating autophagy and angiogenesis [88]. However, despite indications suggesting the value in targeting TFEB for vascular disease treatment, research on the role of TFEB in tumoral angiogenesis remains rare.

### Drug resistance

Multidrug resistance has always been a major problem in cancer treatment, and studying drug resistance mechanisms and strategies to abrogate resistance is aimed at increasing cancer treatment efficacy. Recent studies have shown that lysosomes play important roles in drug resistance, sequestering certain drugs, which are then excreted out of cells through exocytosis. Mechanistically, lysosomes are closely related to P-gp (P-Glycoprotein) trafficking and P-gp-mediated aberrant drug efflux, which is considered a main mechanism underlying drug resistance [106]. TFEB is a core regulator of lysosomal biogenesis and has been proven to promote lysosomal exocytosis [44, 78, 107]. Several studies on TFEB-mediated lysosomal biogenesis and exocytosis leading to drug resistance in different types of cancer have been recently reported (details are discussed below) [93, 108, 109], which renders TFEB targeting a valuable line of inquiry for drug-resistant therapy researchers.

## The function of TFEB and therapeutic strategies for targeting TFEB in several cancer types

In addition to acting on the aforementioned pro-tumorigenesis phenotypes, the function and role of TFEB in various types of tumors have been reported (Table 1).

**Table 1:** TFEB abnormality, functions, and targeted strategies in different types of cancer.

Cancer type	Abnormal of TFEB	Phenotype related	Function and mechanisms	Therapeutic strategies	References
Melanoma	BRAF inhibitor activates TFEB	Drug resistance	BRAF inhibitor activates downstream effector MAPK/ERK which dephosphorylates and activates TFEB, causing chemoresistance via promoting autophagy and lysosomal biogenesis, thus causing resistance to BRAF inhibitor	The combination of BRAF-inhibitors and autophagy-inhibitors avoided BRAF-inhibitor resistance	[66, 101, 116]
Pancreatic ductal adenocarcinoma (PDAC)	Overexpression and the constitutive nuclear translocation	Drug resistance	Overexpression and the constitutive nuclear translocation lead to the higher flux of autophagy in PDAC and support cell growth; TFEB-mediated lysosomal biogenesis enhances drug sequestration and lysosomal exocytosis, and thus promotes drug excretion leading to drug resistance	knockdown of TFEB significantly inhibited glutamine and mitochondrial metabolism; combination of MEKi and lysosome-targeted drugs may improve chemotherapeutic efficacy in PDAC	[45, 108]
Renal cell carcinoma(RCC)	Chromosomal translocation leads to MALAT1-TFEB fusion (also known as ALPHA-TFEB fusion)	Progression	MALAT1-TFEB fusion leads to overexpression of TFEB genes, thus inducing the development of RCCs	Not mentioned	[87, 132, 133]
	TFEB amplification	Invasion and metastasis	High-level TFEB gene copies with frequent PDL1 expression, and VEGFA co-amplification might be responsible for the aggressive characteristic	Not mentioned	[134]
Colorectal cancer (CRC)	The expression level of TFEB is positively correlated with the malignant progression of CRC	Cell proliferation and migration	Overexpression of TFEB predicts a worse prognosis, and TFEB knockdown shows significant inhibition of cell proliferation and migration	Not mentioned	[91]
Breast cancer	TFEB overexpression	Recurrence and drug resistance	TFEB overexpression and intense lysosomal biogenesis tend to present with poor postoperative outcomes and poor prognosis; promote drug resistance by inducing autophagy and thus drug sequestration	Targeting TFEB can increase the efficacy of doxorubicin (DOX) and lower the effective dose of DOX	[105, 109, 146] [150]
Prostate cancer	Overexpression of TFEB	Cell proliferation, tumor progression and migration	The overexpression of TFEB has shown the ability to promote the malignant behavior of tumors in vitro and in vivo; Knockdown of both TFEB and ABCA2 reduced lysosome formation and the expression of matrix metalloproteinases, thus reducing PCA invasion and migration	Not mentioned	[99, 100]
Ovarian cancer	Cisplatin activates TFEB	Drug resistance	Cisplatin activates TFEB by inducing its nuclear translocation, upregulating downstream PD-L1 and PD-L2, forming an immunosuppressive tumor microenvironment, and thus mediate tumor immune escape and drug resistance	TFEB inhibition can increase sensitivity of ovarian cancer cells to cisplatin	[158, 160, 161]
Lung cancer	Overexpression of TFEB	Drug resistance	Overexpression of TFEB is associated with metastasis and poor prognosis of lung cancer	Not mentioned	[95, 164]

### Melanoma

Melanoma is one of the most aggressive human cancers, and its incidence is increasing rapidly worldwide. The main treatment for melanoma is resection in the early stage, which offers the best chance for a cure [110]. Unfortunately, in unresectable malignant melanoma, a low drug response rate and high drug resistance lead to poor prognosis, which has led to the increased interest in identifying the mechanism underlying melanoma tumorigenesis and drug resistance [111, 112].

More than 60 years ago, Paula Hertwig discovered a mouse MITF mutation that subsequently it was proved to affect the development of melanocytes [113]. MIFT was identified as a master regulator of melanocyte growth, and amplified mutations in MIFT and mutations in MiT/TFE family genes have been found in melanoma and are therefore considered oncogenes. Although the high expression and depletion of MITF both inhibit the proliferation of melanoma cells, targeting MITF alone does generate a positive feedback effect; however, combination therapies targeting MITF and BRAF^V600E^ significantly inhibit melanoma growth, exerting an effect on cells that respond poorly to MITF-only depletion [114].

The BRAF^V600E^ mutation has been found in 40%–60% of patients and is considered an oncogenic mutation. Interestingly, the regulation of several cell growth–associated molecules is considered to depend on BRAF^V600E^. BRAF inhibitors have shown optimistic results and are now the standard therapeutic regimen for BRAF^V600E^-positive melanomas [115]. However, drug resistance to BRAF inhibitors results in a poor prognosis in melanoma. This resistance has been attributed to BRAF inhibitor-induced autophagy. BRAF^V600E^ activates the downstream effector MAPK/ERK, which phosphorylates and inactivates TFEB and thus causes autophagy inhibition. BRAF inhibitors in turn promote autophagy via TFEB dephosphorylation and promote autophagy and lysosomal biogenesis, thereby causing chemoresistance. The role played by TFEB has been confirmed by TFEB knockdown, which resulted in a decrease in autophagy. Although activating the “BRAF-TFEB-autophagy‒lysosome” axis promotes tumor progression and chemoresistance, targeting autophagy promotes tumor progression, metastasis and chemoresistance by upregulating TGF-β, which might be a promising therapeutic target in coordination therapies with BRAF inhibitors and autophagy inhibitors [67, 101, 116].

### Pancreatic ductal adenocarcinoma

The incidence of pancreatic cancer is low, but the degree of pancreatic cancer malignancy is high. Pancreatic ductal adenocarcinoma (PDAC) is the most common subtype of this cancer. Due to the lack of specific symptoms and efficient screening, most PDAC patients present with locally advanced or metastatic disease at the time of diagnosis [117].

Autophagy plays a dual role in PDAC, depending on the type and phase of the cancer. Although autophagy has been proven to promote the effects of chemotherapy in several other types of cancer, studies have indicated that increased autophagy is required for or supports cell growth in PDAC [79, 85]. Overexpression and constitutive nuclear translocation of TFEB are found in PDAC cells, which might be the reason for the higher autophagic flux observed in PDAC [79]. Knockdown of MiT/TFE proteins inhibited primary PDAC cell growth, and knockdown of TFE3 and MITF abrogated xenograft PANC-1 tumor growth [86]. Interestingly, knockdown of TFEB led to no significant change in autophagic flux, suggesting an alternative pathway that bypasses signaling after autophagy induction [79] and that TFEB promotes tumorigenesis in an autophagy-independent manner. Kim et al. found that knocking down TFEB significantly inhibited glutamine and mitochondrial metabolism, thus suppressing the growth of PDAC in vitro and in vivo [47]; this finding sheds some light on the therapeutic strategy involving targeted TFEB in PDAC.

PDAC is one of the most aggressive cancers. TGF-β, a regulator of autophagy, in particular, is thought to promote the invasion of pancreatic cancer cells through multiple mechanisms [118]. He R et al. reported that in Smad4-positive PC cells, TGF-β induced TFEB expression through the canonical Smad pathway and thus promoted TFEB-driven autophagy and RAB5a-dependent endocytosis of Itga5, ultimately leading to cancer progression. TFEB knockdown in vivo and in vitro significantly decreased the PDAC cell migration induced by TGF-β, which further supported this hypothesis [119]. Our group further proved that in Smad4-positive PDAC cells, TGF-β-induced autophagy promoted PDAC cell proliferation but inhibited their migration by reducing the nuclear translocation of Smad4. In contrast, TGF-β-induced autophagy inhibited Smad4-negative PDAC cell proliferation but promoted their migration by regulating the MAPK/ERK pathway [118]. These works explored the two autophagy induction mechanisms of SMAD4-dependent and SMAD4-independent PDAC based on distinct genetic contexts and provided a theoretical basis for comprehensive PDAC therapy by targeting autophagy and TFEB.

Among all the gene mutations in PDAC, the oncogene KRAS mutation is the most common, in more than 90% of PDAC patients, and promotes PDAC cell proliferation and prevents their apoptosis, thus inspiring researchers to study drugs targeting the KRAS pathway [108, 117, 120]. The mutant KRAS functions mainly through the activity of the RAF/MEK/ERK and PI3K/AKT pathways. Given that more than 30% of human cancers with mutant RAS respond to no therapy, downstream signaling pathways such as MEK/ERK have been regarded as potential therapeutic targets [121]. However, highly specific MEK inhibitors (MEKis), including trametinib and refametinib, have failed to show clinical benefit in PDAC due to TFEB-mediated lysosomal biogenesis, which increases the drug sequestration and lysosomal exocytosis rates and thus promotes drug excretion. Depletion of TFEB decreased lysosomal biogenesis after MEK inhibition and promoted sensitivity toward MEKi, suggesting that a combination of MEKi and lysosome-targeted drugs may improve chemotherapeutic efficacy in PDAC [108].

### Renal cell carcinoma

Renal cell carcinoma (RCC) accounts for 85% of kidney cancers. RCCs are highly heterogeneous and can be classified into different histopathological subtypes, the most common of which is clear cell RCC [122]. Among renal cell carcinomas, the most genetic changes have been found in RCC, e.g., VHL, 3p, SDHB, SDHC, SDHD, FLCN, TSC1, TSC2, MITF, TFE3, TFEB, FH, MET, and PTEN [123, 124], affecting various pathways such as the oxidative phosphorylation and nutrient and energy metabolism pathways [125], and newly discovered renal tumor subtypes, named on the basis of their characteristic molecular alterations, have been reported (e.g., MIT family translocation carcinomas [126]). As important regulators of catabolic pathways, the roles played by MiT/TFE family proteins in RCCs are complex.

Several TFE-fusion RCCs have been discovered thus far, and they are classified as MiT family translocation RCCs in the 2016 World Health Organization classification [126]. These proteins include TFE3-fusion and TFEB-fusion proteins, which are generated by chromosomal translocation and lead to the overexpression of TFE3/TFEB genes [127]. Various genes fused with TFE3 have been discovered, including PRCC [128], SFPQ [122], NONO [122], ASPSCR1 [122], CLTC [129], ASPL [130], RBM10, and DVL [131], with most located at Xp11.2; cancers harboring these fusion genes are thus called Xp11 translocation RCCs. TFE3-fusion RCCs are relatively aggressive and related to metastasis (reviewed in ref. [122]). TFEB fusion genes are relatively rare, but the number of cases in which they have been identified has gradually increased in recent years, and therefore, a new molecular-driven renal tumor classification named TFEB-altered RCCs was established in the 2022 WHO Classification of Urogenital Tumors [126]. The MALAT1-TFEB fusion protein (also known as ALPHA-TFEB fusion protein) confirmed to be t(6;11)(p21;q12) or t(6;11)(p21.2;q13) causes a rare neoplasm that is most prevalent in young people, in whom it is mostly an indolent disease. This type of RCC rarely metastasizes or recurs after years of follow-up [132, 133], and therefore, it has better prognosis than TFE3-fusion RCCs. Due to a chromosomal translocation, TFEB exons preserved intact gain the powerful MALAT1 promoter, resulting in a significant increase in TFEB mRNA and protein levels and leading to disordered MiT/TFE family transcription factors and metabolic programs, which induces the development of RCCs [87]. In addition, several case reports describe several rare TFEB-fusion genes, including ACTB, NEAT1, KHDBRS2, CADM2, and COL21A1 [131], in which the mRNA of the TFEB transcription factor is highly expressed. In contrast to TFEB-translocation RCCs, TFEB-amplified RCCs are more aggressive and are found mostly in older patients [134]. A high number of TFEB gene copies, usually more than 10 or 20 copies, have been identified via FISH in TFEB-amplified RCCs. Moreover, the PDL1 expression is high, and TFEB and VEGFA co-amplification has been identified in nearly all cases, indicating that these factors might be critical for high invasiveness and metastatic capacity, suggesting that they are promising treatment targets [135].

MITF was previously thought to be associated only with increased genetic susceptibility to RCC and melanoma [136–139], but several MITF-related gene fusions have been identified in recent years [135, 140].

A consensus on the requirements for TFE3 and TFEB analysis in subtype diagnosis for young patients diagnosed with RCC or who present with histological signs suggestive of a translocation RCC subtype was reached at the 2013 ISUP meeting [141], and these criteria have been repeatedly recognized for their diagnostic value [142]. However, for a few study cases, a therapeutic strategy remains to be explored.

### Colorectal cancer

Colorectal cancer (CRC) is characterized by high incidence and high lethality worldwide. The incidence of CRC is higher in developed countries and is increasing globally, becoming the third most common cancer worldwide [143].

Studies have shown that TFEB is generally expressed at lower levels in CRC tissues than in normal tissues. However, the expression level of TFEB was significantly positively correlated with the malignant progression of CRC. TFEB can be used as a prognostic factor, as overexpression of TFEB often predicts a worse prognosis. In addition, TFEB knockdown significantly inhibits cell proliferation and migration, which might indicate that TFEB is a potential therapeutic target, although research on TFEB in this context is lacking [91]. The genetic mutation and regulatory networks of colorectal cancer are complex (reviewed in ref. [144]). Targeting certain factors often leads to a limited effect due to the action of compensatory signaling pathways; therefore, a systematic study of signaling changes in CRC is very important for the formulation of targeted combination therapy strategies [144]. As an important regulator of metabolism, TFEB may promote growth and drug resistance in CRC, but more studies are needed to clarify the roles played by TFEB.

### Breast cancer

The prognosis of breast cancer patients depends on their type and stage. For example, the treatment goal of stage I to III breast cancer is a cure, and the prognosis is relatively good [145]. However, a study reported that in early breast cancer, cases of TFEB overexpression and high lysosomal biogenesis tend to be associated with poor postoperative outcomes and a poor prognosis [105, 146]. Interestingly, other reports showed that overexpression of TFEB in TAMs in the breast cancer microenvironment greatly ameliorated the tumor-promoting gene expression profile of the TAMs (details are explained below) [89], which shows that TFEB plays different roles in different components of the tumor microenvironment at the same time, raising new questions with regard to the targeting TFEB.

Latent and advanced recurrence of disseminated cancer cells for up to 20 years is common in breast and prostate cancer, and from ~20%–40% of breast cancer patients present with recurrence in distant organs, sometimes decades after the initial cancer diagnosis [147, 148]. With the increased development and advances in cancer treatment, early detection and treatment have improved the survival rate of cancer patients, and the course of cancer has been prolonged, but an increase in late recurrence is gradually affecting more patients [149]. Research on the underlying mechanisms through which breast disseminated dormant cancer cells (DDCCs) survive in new environments is rare, but Zangrossi et al. recently published a study showing that the TFEB-lysosomal axis activates DDCCs and promotes relapses. In these experiments, the increase in lysosomal flux was essential for the survival of breast DDCCs, and important targets of TFEB that regulate known lysosomal functions were found to be highly enriched in DDCCs, suggesting an important role for TFEB and its value as a therapeutic target [150].

In addition, although the development of breast cancer-targeted drugs has prolonged survival of patients with breast cancer, chemotherapy such as doxorubicin (DOX) has been particularly important due to the limited use of targeted drugs in triple-negative breast cancer (TNBC), but only 30% of patients treated with DOX achieve remission, and TFEB has been shown to help cells evade DOX-induced apoptosis, which means it can be a therapeutic target in a strategy for lowering the effective dose of DOX [109]. In TNBC, TFEB is hypophosphorylated and is localized to the nucleus in the presence of DOX, and its active form protects the cell from apoptosis. Autophagy has long been thought to promote drug resistance by inducing drug sequestration, and anthracycline treatments have been found to stimulate the autophagy-lysosomal pathway [151–153]. However, interestingly, the apoptosis-preventing and DNA-damage-protective functions of TFEB is evident regardless of the presence of lysosomal inhibitors. The function of TFEB in preventing TNBC apoptosis is not dependent on lysosomes. TFEB knockdown led to the downregulation of homologous recombination (HR) genes, which are involved in homologous DNA repair, and the upregulation of IFN-γ and death receptor signaling, which proved that TFEB can function in a lysosome-independent manner [109].

### Prostate cancer

Prostate cancer (PCa) has a high incidence in men. In contrast to localized PCa, for which the prognosis is relatively good with a survival rate greater than 99%, metastatic PCa is the main cause of PCa deaths [154].

Androgen receptor (AR) is a hormone-regulated factor that plays a crucial role in the development and maintenance of the prostate gland and in the development and progression of prostate cancer [100]. Androgen deprivation therapy is a longstanding and only standard treatment, and it has led to great results [155]. However, metastatic PCa needs to be treated systemically for better efficacy and prognosis, and TFEB may be a novel target for PCa therapy. Studies have shown that TFEB, together with ATG4B, ATG4D, ULK1, and ULK2, is dispensable for androgen-mediated cell proliferation and autophagy. These factors, alone or in combination, may promote prostate cancer progression independently of AR activity [100]. The overexpression of TFEB promoted the malignant behavior of tumors in vitro and in vivo. TFEB promoted the expression of ABCA2 by promoting lysosome formation. ABCA2, an important structure located on the surface of lysosomes and involved in material transport, is very important for lysosome function. Knockdown of both TFEB and ABCA2 reduced lysosome formation and the expression of matrix metalloproteinases, which in turn reduced PCA cell invasion and migration [99].

### Ovarian cancer

For ovarian cancer (OC), platinum is the main treatment option, but platinum resistance occurs, especially when used to treat recurring tumors, leading to a decline in the efficacy of ovarian cancer treatment [156]. Studies have shown that cisplatin (DDP) is enriched in lysosomes, and therefore, DDP resistance is associated with abnormal protein transport and secretion [157].

ATP7B, an ion transporter, was discovered in the liver as a copper-transporting ATPase that protects cells from metal toxicity and is important for liver homeostasis. However, an increasing number of studies have shown that tumor cells leverage ATP7B to detoxify chemotherapy drugs, including platinum, causing drug resistance. A role for TFEB as an important regulator of platinum drug resistance has been confirmed [158]. Although TFEB is reportedly not associated with ovarian tumor proliferation, it has recently been proven to function in cisplatin-resistant OC in a gene expression profile analysis and to be negatively correlated with prognosis in cisplatin-treated OC patients [159, 160]. Studies have shown that in ovarian cancer, TFEB binds to the CLEAR domain in the ATP7B promoter and thus promotes the transcription of ATP7B, enhancing the development of ovarian cancer drug resistance. In contrast, TFEB inhibition results in a decrease in ATP7B expression and increased sensitivity of ovarian cancer cells to cisplatin. These findings demonstrate that TFEB and its downstream target ATP7B are potential targets for platinum-resistant ovarian cancer [93].

Interestingly, studies have shown that cisplatin can in turn act on TFEB, inducing its nuclear translocation, increasing the expression of downstream PD-L1 and PD-L2, forming an immunosuppressive tumor microenvironment, and thus mediating tumor immune escape and drug resistance [160, 161]. Although the mechanism by which cisplatin activates TFEB remains to be explored, undoubtedly, a loop between cisplatin and TFEB promotes drug resistance and tumor progression in ovarian cancer cells, indicating that TFEB is a promising target in drug-resistant ovarian cancer.

### Lung cancer

Lung cancer is the leading cause of cancer death worldwide, and metastatic lung cancer is an important factor in the death of lung cancer patients [162, 163].

A study showed that overexpression of TFEB is associated with metastasis and poor prognosis in lung cancer. TFEB silencing did not affect cell proliferation but inhibited cancer cell migratory ability [95].

Chemotherapy for lung cancer is currently based on a two-drug regimen that includes platinum. However, drug resistance frequently develops. Studies have shown that inhibiting autophagy in lung cancer cells can increase their sensitivity to radiation treatment and chemotherapy [164].

In MiT/TFE family members, DDP treatment leads to a decline in MITF action and dysfunctional lysosomal biogenesis, and knockdown of MITF enhances cell death after DDP treatment [165]. Although few studies on the role of TFEB in DDP resistance in lung cancer have been reported, research on the underlying mechanism of TFEB in lung cancer chemoresistance is worthwhile, especially given that the TFEB in promoting DDP resistance that has been reported in ovarian cancer, as we described above.

## TFEB and the tumor microenvironment (TME)

Specific characteristics of tumor cells, such as a high metabolism rate, create a microenvironment of nutrient deficiency, acidity, hypoxia, and abnormal blood vessel development [166]. This microenvironment promotes tumor growth, supports tumor immune escape, and recruits immune cells to inhibit antitumor function and promote cancer progression [102]. As we explore the tumor microenvironment to a deeper level, it is becoming increasingly clear that understanding and conquering the tumor microenvironment are important goals in tumor treatment.

In a study of the breast cancer tumor microenvironment, up to 50% of breast cancer cells were identified as TAMs [167], and TFEB is an important regulator of TAMs [89]. Tumor cells secrete CSF1 and CCL2 and recruit monocytes/macrophages from the circulatory system, and TAM activity is induced by factors such as IL-10 and TGFβ1 in the tumor microenvironment, driving TAMs to secrete molecules such as arginase 1 (ARG1), IL-10 and TGFβ1, which promote tumor cell survival and immune evasion in the tumor microenvironment [89]. For example, IL-10 directly inhibits T-cell function, and TGFβ1 plays an immunosuppressive role, promoting cancer cell proliferation, inducing the epithelial-mesenchymal transition, and promoting tumor stem cell generation [168, 169]. TFEB regulates TAMs through multiple pathways, such as via the upregulation of suppressor of cytokine signaling 3 (SOCS3) to inhibit M2-like activation of Mφs via STAT3 signaling and the upregulation of PPARγ and autophagy-lysosomal activity to suppress the inflammatory response and degrade HIF-1α, which mediates a response to hypoxia. Interestingly, although the upregulation of TFEB has been detected in many breast cancer cells, as we noted earlier, and upregulation of TFEB in early-stage breast cancer cells often predicts a poor prognosis, and significant TFEB downregulation has been detected in TAMs [89]. Given the complex functions of TFEB in breast cancer and the tumor microenvironment, TFEB is a promising target, as indicated by many studies; however, a systematic study of TFEB in breast cancer and other cancers remains to be performed.

In the study of the tumor microenvironment, inhibiting tumor cells without inhibiting antitumor cells has been a clear aim as T-cell function is important for the clinical efficacy of immunotherapy [170]. The unique characteristics of the tumor microenvironment are not conducive to the antitumor function of T cells and NK cells, but in contrast, T regulatory (Treg) cells, a CD4^+^ T-cell subset with high inhibitory activity, can survive in the tumor microenvironment and hinder antitumor immunity [170]. Studies have shown that TFEB induces CD3epsilon downregulation, is involved in the apoptosis of T cells [171] and is essential for the suppression of Treg cells [172]. TFEB knockout blocked the differentiation of naïve CD4^+^ T cells into Treg cells, and TFEB-specific deletion in mice caused significantly enhanced antitumor effects [172]. The expression of the autophagy-lysosomal pathway was not restricted in TFEB-knockout cells, which indicated that the regulation of TFEB on Treg cells was independent of autophagolysosomes and indicated that TFEB linked nutrients to immunity in T cells; the connection of these pathways suggests new therapeutic targets in the tumor immune microenvironment [172].

In terms of immune regulation in the microenvironment, TFEB is not only closely related to Treg cells but is also a regulator of the immunosuppression of tumor-educated dendritic cells (TEDCs). Dendritic cells (DCs) are specific antigen-presenting cells that integrate various signals and initiate immune responses. The ability of DCs to initiate an immune response or induce cell death is strictly dependent on their maturation state or subpopulation, and tumors hinder the differentiation and maturation of DCs, inhibiting their ability to initiate an immune response [173]. In the tumor microenvironment, TEDCs are involved in tolerance and play an important role in tumor progression [174]. Studies have shown that TFEB plays a key role in regulating antigen presentation by antigen-presenting cells and that upregulation of TFEB leads to enhanced lysosomal proteolytic activity and reduced antigen cross-presentation during LPS-induced DC maturation [175]. In the tumor setting, however, increased expression of TFEB has been detected in LLC cancer cell supernatant-induced TEDCs. TFEB knockdown significantly reduced tumor growth and increased CD11c^+^MHC-II^+^ DCs and CD4^+^ T cells in tumor masses, hindering tumor progression [174].

## Therapeutic strategies discovered thus far and potential future therapies

Since most reports on the cancer-promoting mechanism of TFEB have proven that its effect is achieved by regulating the autophagy-lysosomal pathway, most of the related therapeutic strategies involve drugs targeting the autophagy-lysosomal pathway; these drugs include the lysosomal inhibitor hydroxychloroquine (HCQ), which has shown a good inhibitory effect on tumor growth [176]. The use of small molecules directly targeting TFEB in tumor therapy has not been reported, but due to the conversion of multiple phenotypes of tumor cells after TFEB knockout, small-molecule factors are worth exploring [177]. In addition, by targeting TFEB, the therapeutic doses of other antitumor drugs, such as DOX, are expected to be reduced, making TFEB targeting a novel and promising strategy for use in combination with antitumor drugs [109].

## Conclusion and perspectives

The autophagy-lysosomal pathway plays a variety of roles in human cancer. In addition to degrading toxic waste accumulation and recycling nutrients to promote tumor cell survival, it also participates in tumor immunity, reshapes the tumor microenvironment, promotes vascular development, and promotes tumor progression and metastasis. TFEB is the master regulator of the autophagy‒lysosomal pathway. With the deepening of research, the mechanism by which TFEB acts on various types of cancer cells by regulating the autophagy‒lysosomal pathway has been gradually clarified. Surprisingly, TFEB can act directly on cancer cells in an autophagy-lysosomal independent way. Interestingly, TFEB plays different specific roles in different cancer types and exerts different effects on different cancer subtypes and stages [101].

In general, the important role played by TFEB in the onset and development of various types of cancers is unquestionable, TFEB as a prognostic factor is confirmed in several cancers. However, due to the complexity of TFEB functions, as well as its interaction with other factors and the compensatory effects of certain factors, TFEB-related treatments will not soon be translated into clinical applications. Therefore, research into its role in various types of cancers and exploration of the regulatory system in which it is located is important.
